# Diversity and distribution of amphibians in Romania

**DOI:** 10.3897/zookeys.296.4872

**Published:** 2013-04-30

**Authors:** Dan Cogălniceanu, Paul Székely, Ciprian Samoilă, Iosif Ruben, Marian Tudor, Rodica Plăiaşu, Florina Stănescu, Laurenţiu Rozylowicz

**Affiliations:** 1 University Ovidius Constanţa, Faculty of Natural Sciences and Agricultural Sciences, Al. Universităţii 1, corp B, Constanţa 900470, Romania; 2 Institute of Speleology, Romanian Academy, Calea 13 Septembrie 13, Bucharest 050711, Romania; 3 University of Bucharest, Center for Environmental Research and Impact Studies, 1 N. Bălcescu, Bucharest 010041, Romania

**Keywords:** Amphibia, species distribution, species range, biodiversity data, species richness, rarity

## Abstract

Nineteen species of amphibians inhabit Romania, 9 of which reach their range limit on this territory. Based on published occurrence reports, museum collections and our own data we compiled a national database of amphibian occurrences. We georeferenced 26779 amphibian species occurrences, and performed an analysis of their spatial patterns, checking for hotspots and patterns of species richness. The results of spatial statistic analyses supported the idea of a biased sampling for Romania, with clear hotspots of increased sampling efforts. The sampling effort is biased towards species with high detectability, protected areas, and large cities. Future sampling efforts should be focused mostly on species with a high rarity score in order to accurately map their range. Our results are an important step in achieving the long-term goals of increasing the efficiency of conservation efforts and evaluating the species range shifts under climate change scenarios.

## Introduction

Biodiversity studies have intensified after the Convention on Biological Diversity was signed at the 1992 World Summit in Rio de Janeiro. Despite repeated attempts to halt biodiversity loss, the 2010 targets have not been met (Barbault 2011). The new Aichi Biodiversity Targets provide more detailed and focused targets for 2020, but achieving them requires detailed data and information on biodiversity. Several global scale initiatives have been started to compile the vast biodiversity datasets held in museums and herbaria, publications or data resulting from intensive field surveys e.g., GBIF, EOL (Franklin 2009). The increased amount of biodiversity data available is accompanied by progress in computation that allows not only the proper management of data (Matin et al. 2012) but also its advanced and precise analysis (Reese et al. 2005). Basic knowledge of the species distributions within a region is required for a proper management of biodiversity, e.g., to predict species extinction under habitat loss, to understand the potential impacts of climate change on biodiversity, to prioritize conservation efforts and design conservation areas (Margules and Pressey 2000, Primack 2010).

Mapping species distribution data is a popular and successful way of assembling and presenting spatial information on a variety of organisms, including amphibians (Gasc et al. 1997). While most mapping projects end with the production of an atlas, the occurrence data can be further analyzed or transformed (e.g., Araújo 2004, Arntzen 2006). The species distribution maps allow answering fundamental questions in ecology and macroecology, such as patterns of abundance, rarity, richness, turn-over and assemblage composition at different spatial scales (Elith et al. 2010, Baselga et al. 2012, Denoël 2012). Moreover, databases of species occurrences have been used in conservation planning, investigation and testing the performance of different methodologies (Jones-Farrand et al. 2011) especially the selection of protected areas (Williams et al. 2000, Araújo et al. 2004), providing a framework for survey design, assessing species-environment associations, and modeling species distribution (Sillero et al. 2005, Popescu and Gibbs 2009). Comprehensive data on species occurrences are required for the identification of threatened taxa. Two of the five sets of criteria used by IUCN (Red List Criteria version 3.1, IUCN 2001) are based on geographic range and shifts in range (i.e., Extent of Occurrence and Area of Occupancy). Making the existing information available to the broader scientific community for use in conservation, education and sustainable development is a priority. Nevertheless, the data need to fulfill minimal criteria to be exploitable. For example Stork et al. (1996) cited three reasons for which actions affecting biodiversity and conservation are based on inadequate information: (1) the data necessary for informed decision-making are unavailable, incomplete, or unreliable, (2) the data are not presented in a format that policy-makers and managers can use, and (3) the data are incorrectly interpreted.

Eastern European countries do not usually provide quality distribution data due to less uniform and intensive recording effort (Williams et al. 2000). This is also valid for Romania (Iojă et al. 2010) where, after publishing the volume on Amphibians in the series Fauna of Romania (Fuhn 1960), only two papers updated the distribution of amphibians using the UTM 10 **×** 10 km grid (i.e., Cogălniceanu 1991, Cogălniceanu et al. 2000), and few up-to-date reviews are available on the distribution of single species (e.g., Sas et al. 2008, Sas 2010) or for the herpetofauna of given areas (e.g., Ghira et al. 2002, Covaciu-Marcov et al. 2006, Székely et al. 2009). Despite a significant increase in the inventory effort in Romania, mostly in the years after 2000, there is no available distribution database, nor a published atlas. Considering the needs for an updated overview of diversity and distribution of amphibians in Romania, we compiled a database with species occurrences, mapped the amphibian species occurrences in Romania, and analyzed the spatial patterns of the data.

## Methods

### Mapping species occurrences

We used occurrence records from four major sources: published data, museum collections, personal communications from specialists, and our own unpublished field data. The records were stored and managed in a Microsoft Access database and later imported in a GIS environment as geodatabase. For spatial representation we used the Universal Transverse Mercator (UTM) grid system which is the most frequently used cartographic framework for mapping species distributions (Franklin 2009). The occurrences were aggregated and georeferenced to the UTM 5 **×** 5 km grid system.

The distribution records with a spatial resolution ≤ 25 km^2^ were georeferenced and assigned the corresponding alphanumeric UTM 5 **×** 5 km grid cell code. The occurrences with a spatial resolution > 25 km^2^ were assigned in Google Earth (Google Earth v7.0.2, Google Inc., CA) to a single grid cell, based on expert knowledge of the species’ habitat requirements (Scott et al. 2002, Franklin 2009). The occurrences were classified as old, if recorded before 1990 and recent, if after 1990, based on the year of observation. If the year of observation was not mentioned in the publication, we subtracted 3 to 5 years from the date of publication.

Several records were not used for mapping species distributions when they could not be referenced to a specific locality or toponymy (e.g., distribution records assigned to mountain ranges, geographical provinces or hydrographic basins, without a finer scale reference), or unspecified taxa within a genera, while other records were doubtful or erroneous (for a detailed description of errors see Chapman 2005). A final visual check was done on maps that allowed tracking erroneous records. We identified two sources of errors: (1) author related errors (e.g., errors in identification or transcription errors), and (2) our transcription errors. Most frequent misidentification involved the hybridizing species (*Bombina bombina* and *Bombina variegata*, *Triturus cristatus* and *Triturus dobrogicus*), the brown frogs (*Rana dalmatina*, *Rana temporaria* and *Rana arvalis*), water frogs (*Pelophylax* kl. *esculentus*), and juveniles or larvae. Most out-of-range reports, sometimes based on a single individual, were not considered until further confirmation.

Three pairs of amphibian species hybridize in Romania: *Bombina bombina* with *Bombina variegata*, *Lissotriton vulgaris* with *Lissotriton montandoni*, and *Triturus cristatus* with *Triturus dobrogicus*. In most cases, occurrences were assigned to a hybrid based on the analysis of morphological features. The grid cells in which hybrids occurred were mapped distinctly for both parental species. The data used for mapping the hybrid zones varied in quality: while some reports refer to “hybrids” or “intergrades”, others were identified based on reports of both parental species occurring in the same grid cell. Since the large water frogs of the *Pelophylax* complex (*esculentus* and *ridibundus*) are difficult to distinguish (Pagano and Joly 1999), we decided to consider *Pelophylax lessonae* separately, while the other two taxa (*esculentus* and *ridibundus*) were mapped together (similar to Cogălniceanu and Tesio 1993). *Lissotriton vulgaris* and *Lissotriton vulgaris ampelensis* were plotted with different symbols as the second is a taxon of European conservation interest, i.e., protected under the European Habitats Directive (92/43/EEC 1992). In all other analyses we considered them as a single taxonomic unit, i.e., *Lissotriton vulgaris*.

### Spatial patterns analysis

To identify the potential bias in sampling effort, we first counted the number of amphibian records per grid cell. Then, we used Global Moran’s I to assess the general trend of spatial autocorrelation in occurrences across the entire country. If the Moran’s I test is statistically significant the spatial pattern of amphibian records per grid cell can be spatially clustered (*Z* > 0), dispersed (*Z* < 0) or random (*Z* = 0) (ESRI 2012). To further evaluate the local spatial association in sampling effort, we used Getis Ord Gi* spatial statistic (Ord and Getis 1995). Thus, we identified clusters of grid cells where the sampling effort is significantly higher (i.e., hotspots of occurrences) or lower (i.e., coldspots of occurrences) than expected by random chance. We set up the distance threshold to 7100 m as to include eight neighbors of a grid cell (Getis and Ord 2010).

The mean altitude of 5 **×** 5 km grid cells was extracted from SRTM data (Jarvis et al. 2008) in order to review the altitudinal range of the species. The grid cells exceeding the Romanian boundary were excluded from the analysis.

We calculated the Extent of Occurrence (EOO) as a 100% minimum convex polygon using the state boundary as a mask, in order to remove the areas outside Romania, where no sampling was accounted. We estimated the Area of Occupancy (AOO) as the total area of 5 **×** 5 km grid cells where a species was reported (IUCN 2001).

To map species richness we aggregated the data to a spatial resolution of 50 **×** 50 km UTM grid cells. Mapping the species richness at a coarser resolution reduced the potential bias in sampling effort and allowed a better understanding and visualization of regional patterns (Graham and Hijmans 2006). To compare the individual species range size with the country area, we computed a rarity index for the same 50 km grid resolution. The index takes value between 0 (i.e., widespread species) and 100 (i.e., absent species) (Romano et al. 2012).

All spatial analyses were performed in ArcGISDesktop 10 (ESRI, CA), with *α* = 0.05.

## Results

### Species occurrences

**Figure 1.**
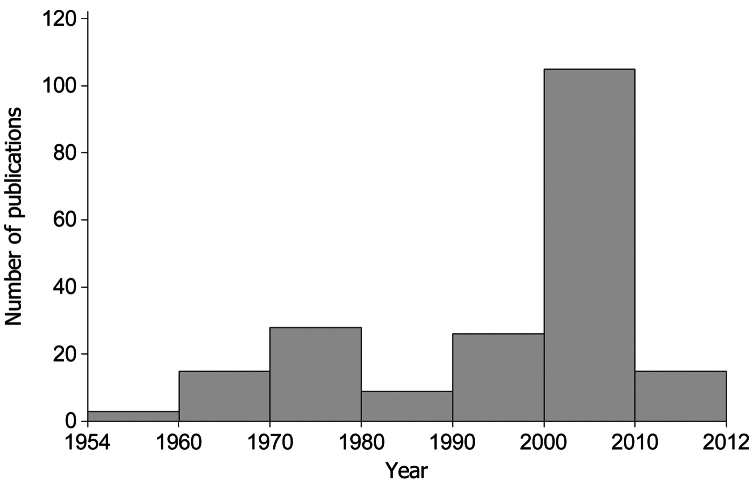
Number of publications containing amphibians’ distribution data (1954–2012).

In total, 26779 amphibian occurrences were compiled and stored in a geodatabase. We collected 20868 records from 201 published papers (Appendix 1). Most papers (59%) were published after 2000 (Fig. 1). We compiled further 5381 unpublished records from our own field surveys and 530 museum collection records. The majority of occurrences were dated after 1990 (89%) and only 11% represented old records collected before 1990. The detailed occurrence statistics for each species are presented in Table 1. Our database increased the average number of amphibian records per 100 km² compared with GBIF dataset (http://data.gbif.org, accessed 15.02.2013) from 0.123 in the GBIF dataset to 11.2 in our database (Table 2).

**Table 1.** The occurrences and measures of amphibian species range in Romania. Extent of Occurrence (EOO) was estimated as 100% minimum convex polygon, and Area of Occupancy (AOO) as the total area of 5 **×** 5 km UTM cell containing species records. Since not all grid cells matched the 25 km² area, the computed AOO is not a multiple of this value.<br/>

**Species**	**Total number of records**	**New records<br/> (after 1990)**	**Total number of UTM5 cells**	**EOO<br/> (km^2^)**	**AOO<br/> (km^2^)**	**Rarity index**
*Salamandra salamandra*	1200	1033	775	132910	29769	50.5
*Triturus cristatus*	1639	1404	436	190721	40763	31.7
*Triturus dobrogicus*	209	180	145	189130	4974	75.6
*Ichtyosaura alpestris*	768	629	962	108965	19161	59.3
*Lissotriton vulgaris*	2114	1846	1220	228999	52377	17.1
*Lissotriton montandoni*	569	429	284	56773	14163	78.9
*Bombina bombina*	1720	1569	936	235347	42033	21.9
*Bombina variegata*	3116	2811	1445	151826	77580	43.1
*Pelobates fuscus*	618	518	399	230794	14957	40.7
*Pelobates syriacus*	153	122	59	48292	3546	87.9
*Bufo bufo*	2128	1968	1291	223802	52561	24.4
*Bufo viridis*	2006	1791	1299	235801	48864	12.2
*Hyla arborea*	1801	1676	1156	233887	44265	11.4
*Rana dalmatina*	2027	1833	1270	225426	49982	25.2
*Rana arvalis*	351	270	215	88423	8638	69.1
*Rana temporaria*	1806	1614	1020	132586	44930	49.6
*Pelophylax lessonae*	203	195	108	190870	4867	73.2
*Pelophylax* kl. *esculentus*	4351	3981	1928	236441	105855	5.7
**Total**	**26779**	**23869**	**-**	**-**	**-**	**-**

**Table 2.** The bias in amphibian species occurrence data for several European countries extracted from GBIF (downloaded on January 30, 2013).<br/>

**Country**	**GBIF records**	**Country area (km^2^)**	**Average number of records per 100 km^2^**
Luxembourg	8119	2666.7	304.461
United Kingdom	101305	260565.6	38.878
Spain	37764	544571.2	6.934
France	24928	570427.7	4.370
Poland	6753	314347.5	2.148
Hungary	160	93374.6	0.171
**Romania**	**293**	**237318.9**	**0.123**
Ukraine	737	600856.2	0.122
Turkey	452	794961.2	0.056
Greece	61	133552.6	0.045

### Spatial patterns in species occurrences

**Figure 2.**
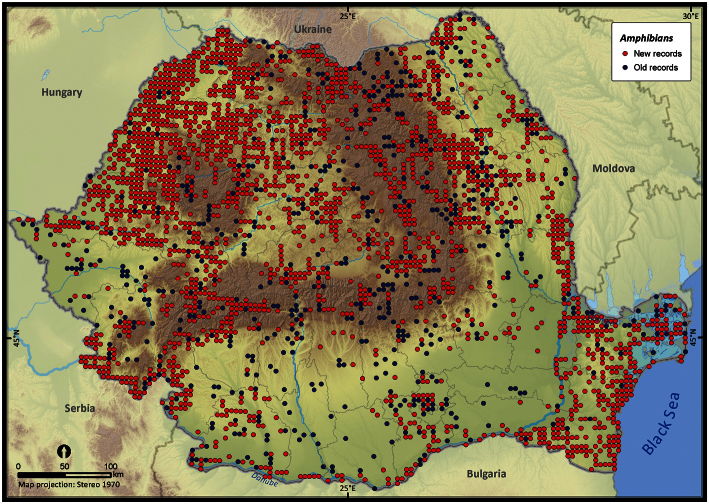
Amphibian occurrences in Romania. Records reported before 1990 were plotted as old records whereas those reported after 1990 were considered new records.

**Figure 3.**
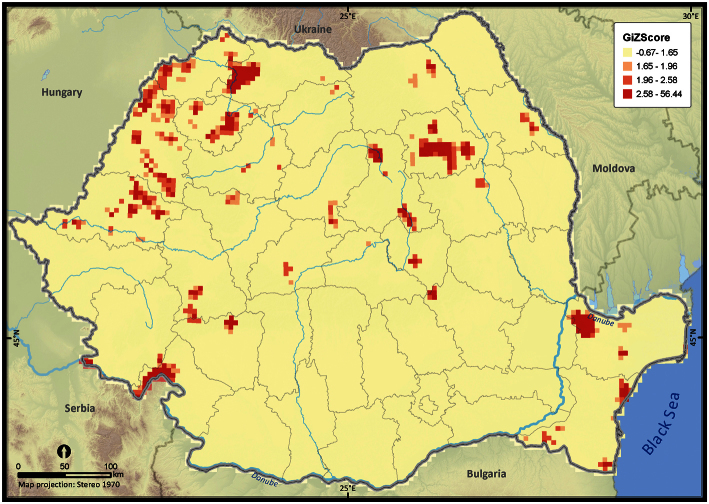
Hotspots of sampling efforts within Romania. The *p* value is < 0.05 when Z scores take values between 1.65 and 56.44, suggesting a highly clustered pattern in the number of amphibian occurrences per UTM 5 × 5 grid cell.

From a total of 9977 UTM 5 × 5 km grid cells covering the Romanian territory, 3013 grid cells (i.e., 30.1%) contain amphibian sightings (Fig. 2). The number of records per cell is highly skewed (skewness = 31.25). Global Moran’s I test revealed a clustered pattern in the number of amphibian records per cell (*Z* = 66.91, *p* < 0.001), thus suggesting a strong bias in nationwide sampling effort. Since 73% of the country surface had ≤ 2 records per cell, the Getis Ord Gi* spatial statistic did not identify the coldspots of sampling efforts as being statistically significant, at the specified threshold distance of 7100 m, but it effectively identified the hotspots of sampling effort (Fig. 3). Among these hotspots, the Măcin Mountains National Park had the highest sampling effort (mean *Z* = 24.36). Other hotspots of sampling effort were the Jiului Gorge National Park (mean *Z* = 2.47) and the Iron Gates Natural Park (mean *Z* = 1.68). There was an obvious trend of clustering the number of records in close proximity to cities (e.g., Iaşi, Piatra Neamţ, Oradea and Satu Mare) (see Fig. 3).

**Figure 4.**
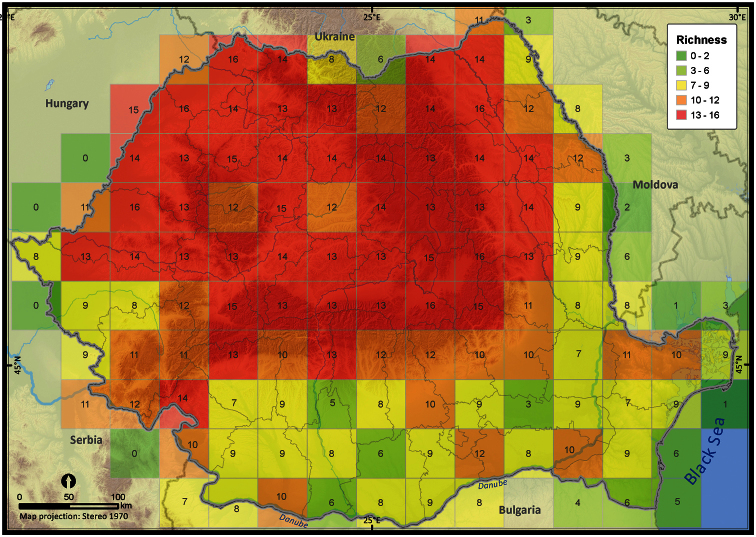
Amphibian species richness within Romania at a 50 **×** 50 km grid resolution.

The species richness represented on the 50 × 50 km grid, excluding the incomplete grid cells, ranged from 3 to 16 (Fig. 4). Individual species (i.e., excluding *Pelophylax* kl. *esculentus*) had different distribution patterns with the Extent of Occurrence ranging from 48292 km^2^ (*Pelobates syriacus*) to 235801 km^2^ (*Bufo viridis*), the Area of Occupancy ranging from 3546 km^2^ (*Pelobates syriacus*) to 77580 km^2^ (*Bombina variegata*), and the rarity index ranging from 75.6 (*Triturus dobrogiscus*, the rarest species) to 11.4 (*Hyla arborea*, the widespread species) (see Table 1).

Taking into account the mean altitude of 5 × 5 km occupied grid cells, the altitudinal gradient of amphibians varied between 0 and 2007 m, with *Ichtyosaura alpestris* and *Rana temporaria* occurring over 2000 m (Fig. 5). The amphibian species occurrence maps in Romania are presented in Figs 6–23. Several species are widespread within the entire country (e.g., *Lissotriton vulgaris*, *Bufo viridis*) while others are restricted only to higher slopes (e.g., *Lissotriton montandoni*, *Ichtyosaura alpestris*, *Salamandra salamandra*), to plains (e.g., *Pelobates syriacus*) or floodplains (e.g., *Triturus dobrogicus*).

**Figure 5.**
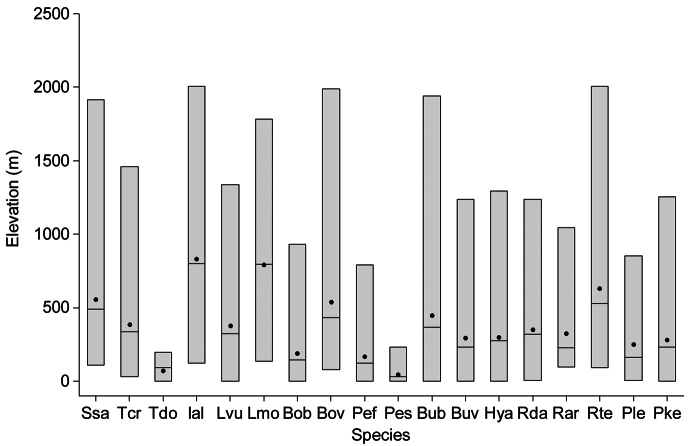
Altitudinal distribution of amphibian species in Romania (dot – mean, horizontal line – median, vertical bar – range, Ssa – *Salamandra salamandra*, Tcr – *Triturus cristatus*, Tdo – *Triturus dobrogicus*, Ial – *Ichtyosaura alpestris*, Lvu – *Lissotriton vulgaris*, Lmo – *Lissotriton montandoni*, Bob – *Bombina bombina*, Bov – *Bombina variegata*, Pef – *Pelobates fuscus*, Pes – *Pelobates syriacus*, Bub – *Bufo bufo*, Buv – *Bufo viridis*, Hya – *Hyla arborea*, Rda – *Rana dalmatina*, Rar – *Rana arvalis*, Rte – *Rana temporaria*, Ple – *Pelophylax lessonae*, Pke – *Pelophylax* kl. *esculentus*).

**Figure 6.**
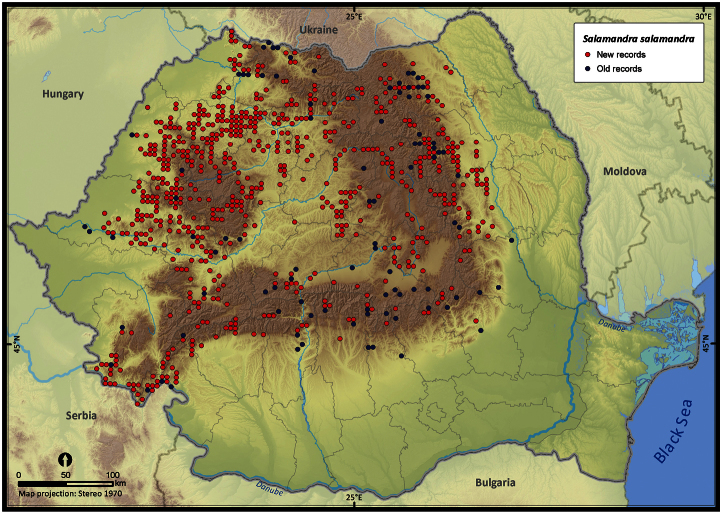
*Salamandra salamandra*.

**Figure 7.**
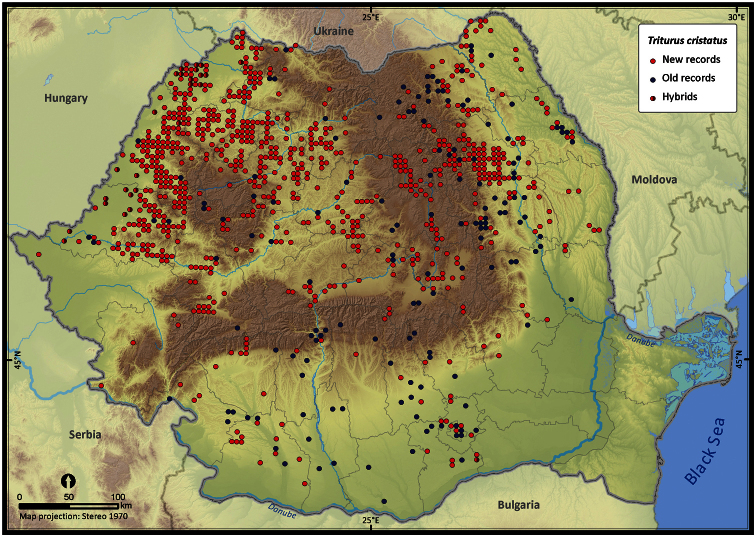
*Triturus cristatus*.

**Figure 8.**
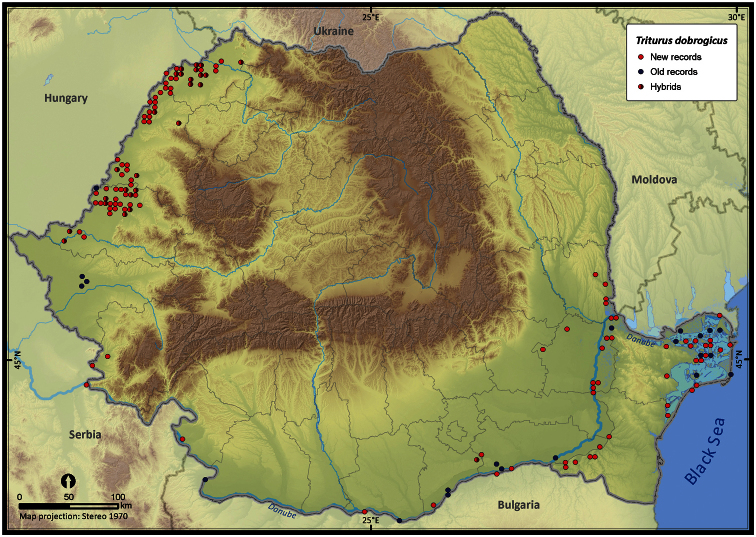
*Triturus dobrogicus*.

**Figure 9.**
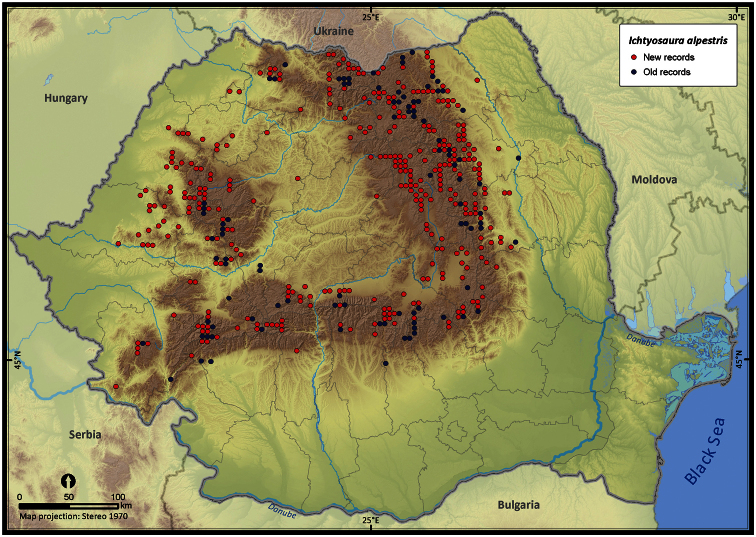
*Ichtyosaura alpestris*.

**Figure 10.**
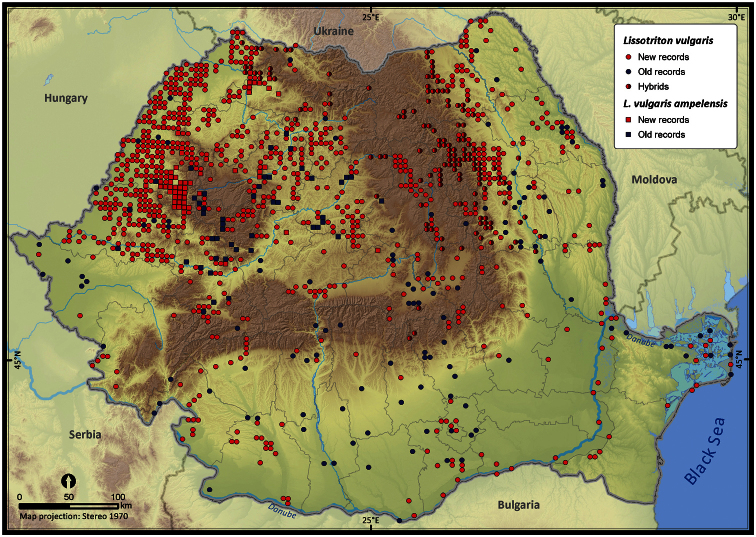
*Lissotriton vulgaris*.

**Figure 11.**
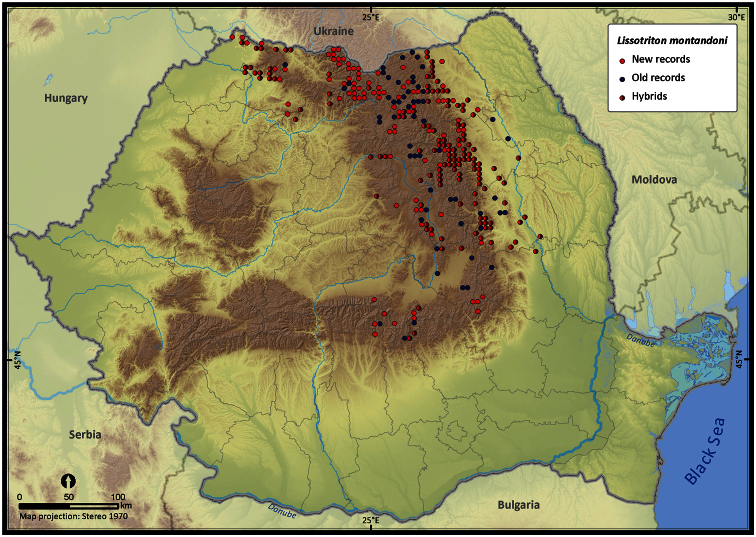
*Lissotriton montandoni*.

**Figure 12.**
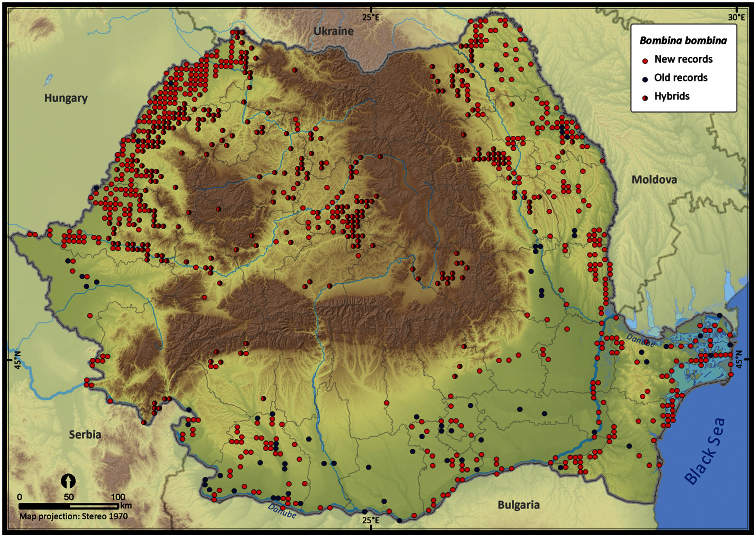
*Bombina bombina*.

**Figure 13.**
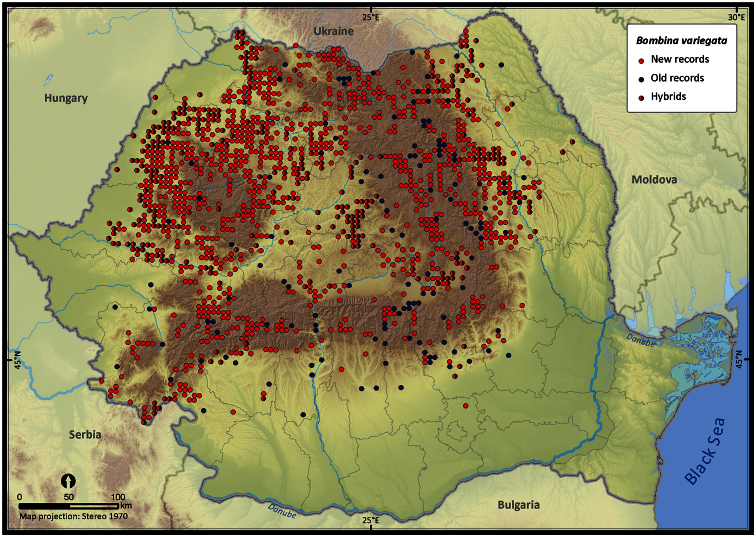
*Bombina variegata*.

**Figure 14.**
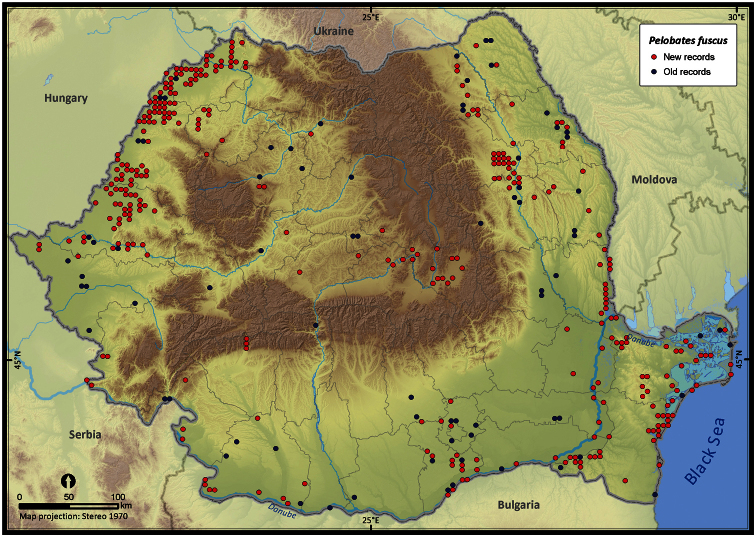
*Pelobates fuscus*.

**Figure 15.**
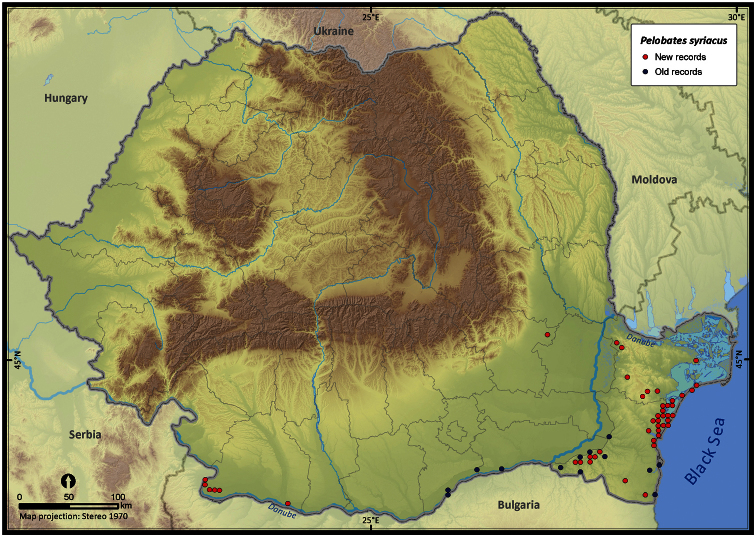
*Pelobates syriacus*.

**Figure 16.**
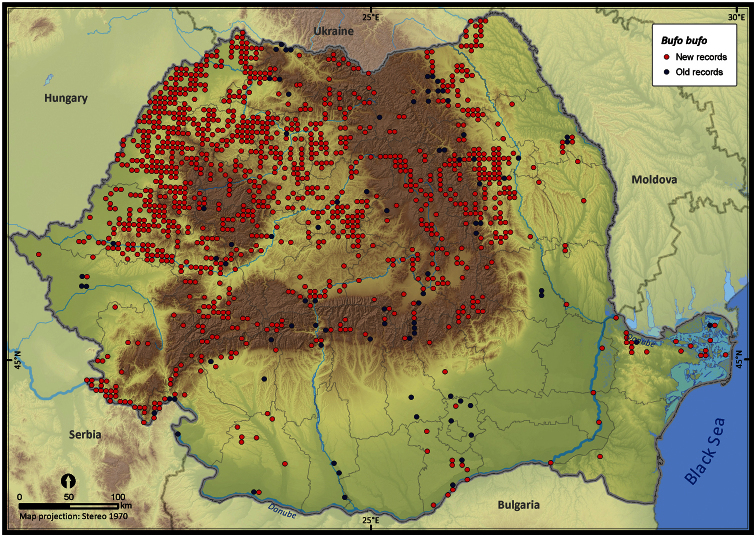
*Bufo bufo*.

**Figure 17.**
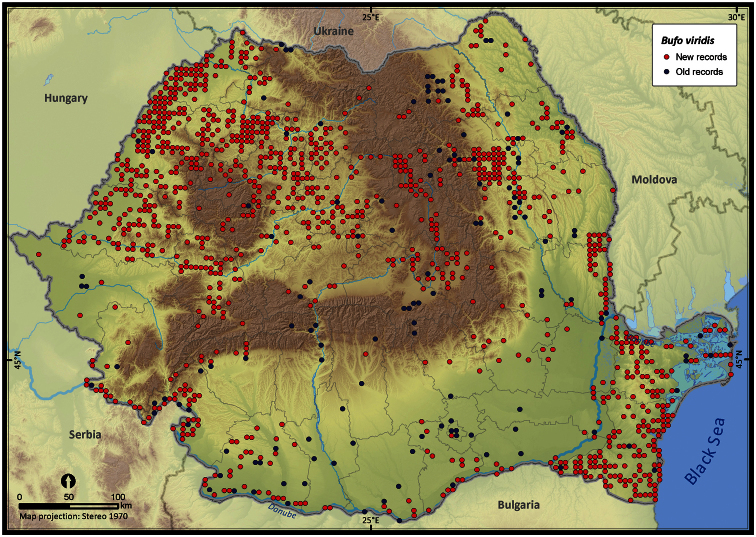
*Bufo viridis*.

**Figure 18.**
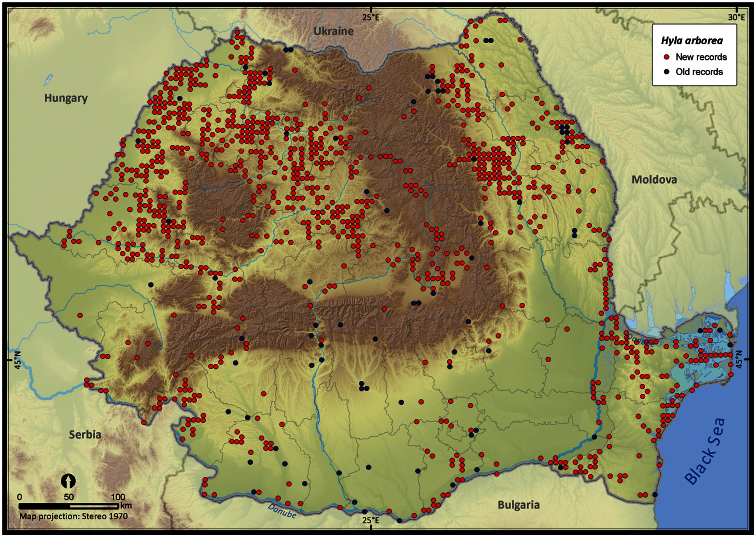
*Hyla arborea*.

**Figure 19.**
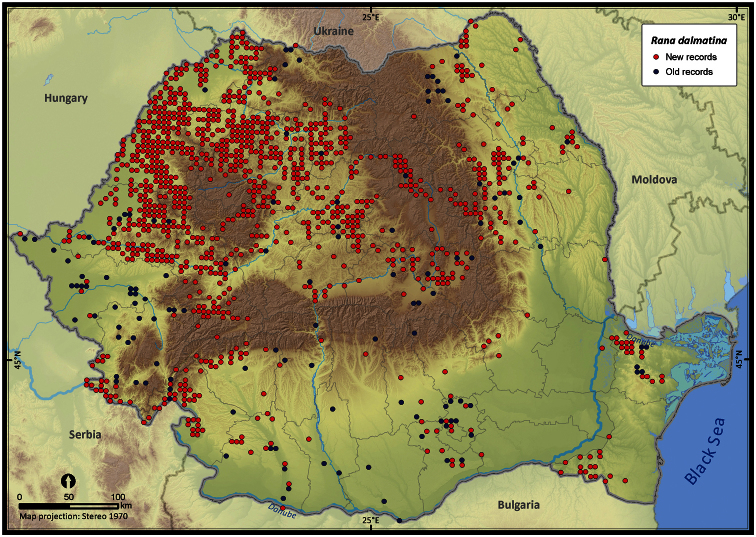
*Rana dalmatina*.

**Figure 20.**
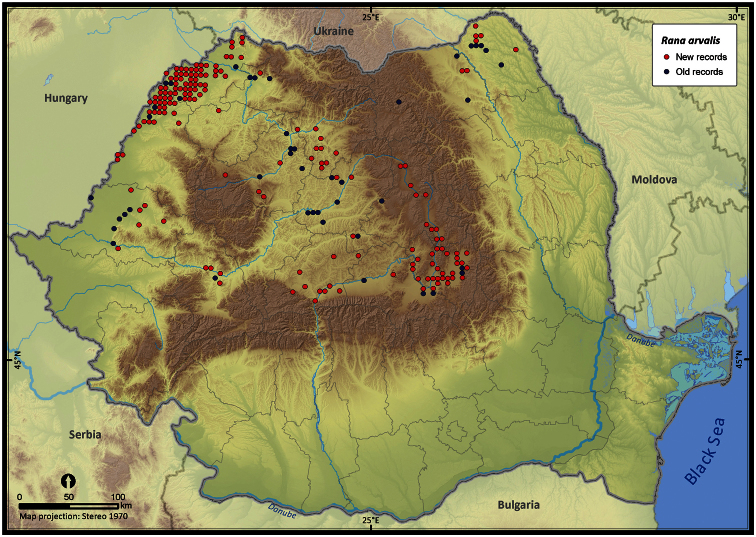
*Rana arvalis*.

**Figure 21.**
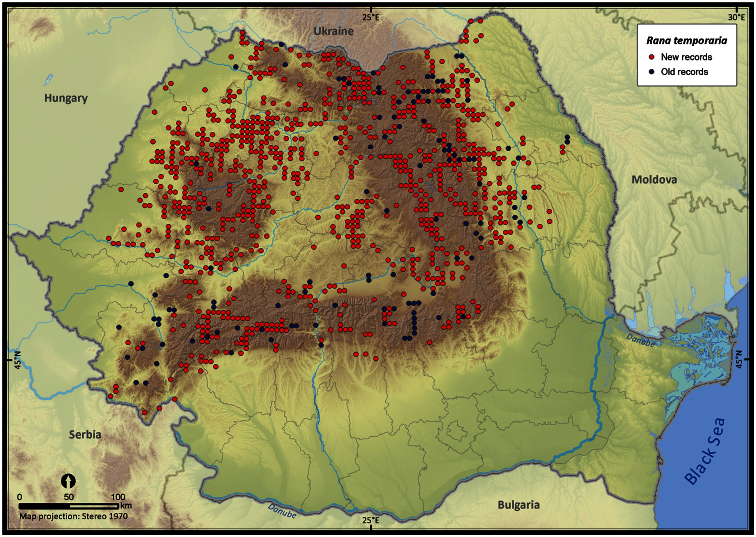
*Rana temporaria*.

**Figure 22.**
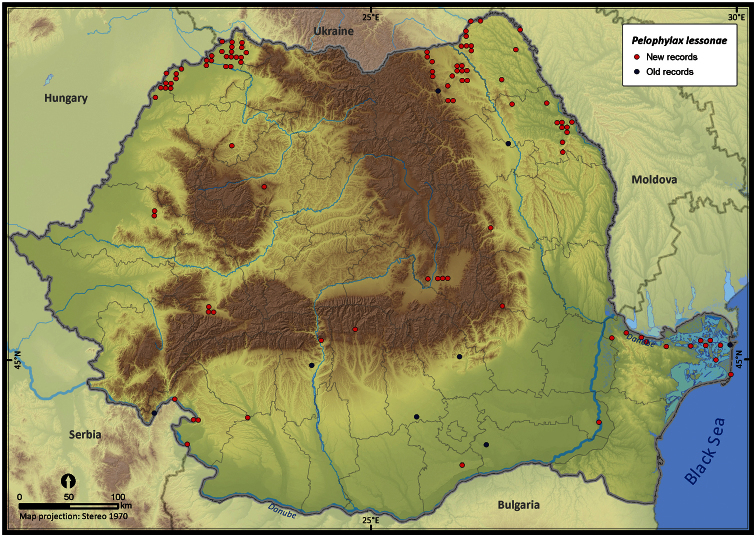
*Pelophylax lessonae*.

**Figure 23.**
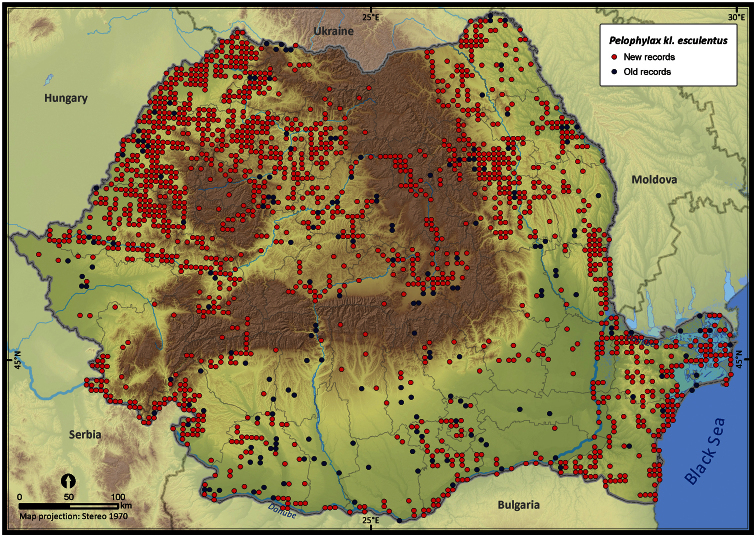
*Pelophylax* kl. e *sculentus* and *ridibundus*.

## Discussion

By compiling the current database of amphibian distribution in Romania, we made available comprehensive distribution maps for further analyses, and provided the first nationwide spatial statistical analysis of the amphibians’ occurrence records.

The number of occurrences varies among species, the largest number of records (i.e., 16%) being that of *Pelophylax* kl. *esculentus*, a taxon including two species (*Pelophylax esculentus* and *Pelophylax ridibundus*). The altitudinal range of *Pelophylax* kl. *esculentus* varies between 0 and 1255 m. The highest number of single taxa occurrences belongs to *Bombina variegata* (i.e., 12%), a species with a broad altitudinal gradient (80-1988 m). Despite the large number of occurrences, *Bombina variegata* is not the most common species in Romania (rarity index = 43.1), yet it has a high detectability (i.e., easy to recognize, is active during the day and occurs mostly on the road-side temporary ponds (Hartel 2008) ). The next species with more than 2000 records in our database are *Bufo bufo*, *Lissotriton vulgaris*, *Rana dalmatina*, and *Bufo viridis*, species with a wide altitudinal range, inhabiting both forests and open habitats (Cogălniceanu et al. 2000) (see Fig. 5).

The number of occurrences per species is negatively correlated with the rarity index (Spearman *rho* = -0.8, *p* < 0.001), indicating that the range of many species is scarcely known. For some species, the large number of occurrences is not reflected in a high rarity score. However, the ranges of *Bombina variegata* and *Rana temporaria* seem to be accurate since they have a high number of occurrences, but a moderate rarity score. At the species level, the future sampling effort should be focused mostly on species with a high rarity score in order to accurately map their range.

A general feature of a research study is that data are considered as a means to an end (i.e., publication), and are therefore treated as consumables (Michener 2000). This feature seems to affect our mapping project. We identified two major faults in our long-term nationwide sampling effort: (1) the bias in data collection, and (2) the lack of standards in publication. Since much of the occurrence data were collected opportunistically rather than systematically, large spatial biases occurred (e.g., Botts et al. 2011, Chapman 1999, Williams et al. 2002). For example, a similar study that conducted an analysis of the data quality obtained in the South African Frog Atlas Project showed a significantly higher sampling intensity near cities and roads and some protected areas (Botts et al. 2011). This geographic bias limits the applicability of an atlas for fine scale conservation planning. The results of our spatial statistic analyses supported the idea of a biased sampling within Romania, with clearly delineated hotspots of sampling effort. Our analysis showed further that the species richness is higher in those areas where the three pairs of species hybridize and lower in the agriculture-dominated plains.

The incomplete and biased species inventory in Romania may have several causes: difficult access due to low road density, complex landscape (with 15% of the territory above 800 m), limited funds available for large-scale inventory and monitoring projects, and lack of institutional support. For instance, no species distribution databases are publicly available at the Romanian Ministry of the Environment (Melinte et al. 2004).

The low quality of biodiversity datasets and the lack of standards in publication and data management limit their usefulness (Palmer and Richardson 2012). There is a growing interest in standards for taxonomic information (e.g., Taxonomic Databases Working Group 2007) and hopefully the quality of datasets will improve in the near future. The geospatial database and outputs presented in this paper as occurrence records fill a gap in our knowledge. In addition, our mapping exercise may allow future predictions of species range shifts under climate change scenarios, as well as prioritization of conservation efforts and identification of important conservation areas for amphibians.

### Acknowledgements

We are grateful to the following persons for sharing their distribution data with us: Dr Arntzen Jan, Dr Bănărescu Petru, Dr Bereş Iosif, Buhaciuc Elena, Dr Gâldean Nicolae, Dr Hartel Tibor, Dr Kyek Martin, Dr Pârvulescu Lucian, Dr Oţel Vasile, Dr Skolka Marius, Sós Tibor, Talbot Neil, and Tallowin Oliver. We thank Dr Franco Andreone, Dr Antonio Romano, and an anonymous reviewer for their constructive comments on the paper. This work was supported by two grants of the Romanian National Authority for Scientific Research, CNCS-UEFISCDI, project number PN-II-RU-TE-2011-3-0183 (principal investigator Laurenţiu Rozylowicz) and CNCS-UEFISCDI, project number PN-II-ID-PCE-2011-3-0173 (principal investigator Dan Cogălniceanu).

## Appendix

Publications used to compile distribution of amphibian species native to Romania. (doi: 10.3897/zookeys.296.4872.app) File format: Microsoft Word document (doc).
