# Supplementary material for: Diversity and distribution of amphibians in Romania
Source: Zookeys. 2013 Apr 30;(296):35–57. doi: 10.3897/zookeys.296.4872 (PMC3689111; doi:10.3897/zookeys.296.4872)
Supplement: Supplementary file 1 — Publications used to compile distribution of amphibian species native to Romania. (doi: 10.3897/zookeys.296.4872.app) File format: Microsoft Word document (doc). [file ZooKeys-296-035-s001.doc]

**Appendix 1**

**Publications used to compile distribution of amphibian species native to Romania**

Andrei M (1997) Note on the herpetofauna of the Maramureş (Romania). Travaux du Muséum National d'Histoire Naturelle "Grigore Antipa" 37: 129-133

Andrei M (2002) Contributions to the knowledge of the herpetofauna of southern Dobruja (Romania). Travaux du Muséum National d'Histoire Naturelle "Grigore Antipa" 44: 357-373

Ardelean G, Trifonov P (2002) Vertebratele din Ţara Făgăraşului. Satu-Mare, Studii şi Comunicări, Seria Ştiinţele Naturii 2/3: 197-204

Băcescu M (1954) *Pelobates syriacus* balcanicus Karaman, o broască nouă pentru fauna Republicii Populare Romîne. Comunicările Academiei RPR 4: 483-490

Băcescu M (1959) La faune relique des sources se trouvant a l'extremite ouest du Tekirghiol et le probleme s'une reservation naturelle dans cet endroit. In: Lucrările Sesiunii Ştiinţifice a Staţiunii Zoologice Marine "Prof I Borcea"-Agigea, 15-17 sept 1956. Iaşi, 1-7 pp.

Bănăduc D (2004) Colecţia de amfibieni a Muzeului de Istorie Naturală Sibiu. Studii şi Comunicări-Ştiinţele Naturii, Muzeul Brukenthal Sibiu 29: 215-219

Bazilescu E, Sorescu C, Cruce M, Popescu M (1980) Catalogul sistematic al colecţiilor de vertebrate din Muzeul Olteniei. Oltenia, Studii şi Comunicări, Ştiinţele Naturii: 311-401

Béres I (1997) Contribuţii la cunoaşterea faunei amfibienilor şi reptilelor din Depresiunea Maramureşului şi protecţia lor. Nymphaea Folia naturae Bihariae 23/25: 151-154

Bogdan HV, Badar L, Goilean C, Boroş A, Popovici AM (2012) Population dynamics of *Triturus cristatus* and *Lissotriton vulgaris* (Amphibia) in an aquatic habitat from Banat region, Romania. Herpetologica Romanica 6: 41-50

Borcea M (1980) Consideraţii asupra faunei de vertebrate din rezervaţia Hanu Conachi şi împrejurimi. Ocrotirea naturii în Moldova. Iaşi, 140-145

Borcea M (1983) Fauna de amfibii şi reptile din Munţii Rodnei. Prezentare zoogeografică. Rezervaţia Pietrosul Rodnei la 50 ani. Baia-Mare, 120-127

Borcea M, Vancea S (1981) Observaţii asupra amfibiilor şi reptilelor din zona Cheile Bicazului-Lacu Roşu în perioada 1978-1979. Ocrotirea naturii şi a mediului înconjurător 25: 91-95

Chiriac E, Malcoci E (1968) Contribuţii la cunoaşterea helminţilor şi amfibienilor din regiunea Iaşi. Analele Ştiinţifice ale Universităţii "Al I Cuza" Iaşi, secţiunea III, Biologie 14: 301-308

Cicort-Lucaciu AS, Covaciu-Marcov SD, Bogdan HV, Sas I (2012) Implication upon herpetofauna of a road and its reconstruction in Carei Plain Natural Protected Area (Romania). Ecologia Balkanica 4: 99-105

Cogălniceanu D (1991) A preliminary report on the geographical distribution of amphibians in Romania. Revue Roumaine de Biologie Serie de Biologie Animale 36: 39-50

Cogălniceanu D, Ghira I, Ardeleanu A (2001) Spatial distribution of herpetofauna in the Retezat Mountains National Park–Romania. Biota 2: 9-16

Cogălniceanu D, Tesio C (1993) On the presence of *Rana lessonae* in Romania. Amphibia-Reptilia 14: 90-93

Covaciu-Marcov SD (1999) Contribuţii la studiul herpetofaunei dealului Şomleu. Analele Universităţii din Oradea, Fascicula Biologie 6: 175-190

Covaciu-Marcov SD (2001) Contribuţii la cunoaşterea răspândirii speciei *Triturus alpestris* Laurentus 1768 în judeţul Bihor, România. Analele Ştiinţifice ale Universităţii de Stat de Medicină şi Farmacie "Nicolae Testemiţanu" 1: 37-41

Covaciu-Marcov SD (2002) Date preliminare privind herpetofauna Văii Ierului. Satu-Mare, Studii şi Comunicări, Seria Ştiinţele Naturii II-III: 191-196

Covaciu-Marcov SD, Bogdan HD, Paina C, Toader S, Condure N (2008) The herpetofauna of the north-western region of Bihor County, Romania. Biharean Biologist 2: 5-13

Covaciu-Marcov SD, Cicort-Lucaciu AS (2009) Big and nonmethamorphic *Triturus cristatus* larvae from north-western Romania. Biharean Biologist 3: 87-89

Covaciu-Marcov SD, Cicort-Lucaciu AS, Dimancea N (2009) What do the newly discovered *Lissotriton montandoni* (Caudata, Salamandridae) populations from Iezer Mountains, Romania, have to say about the species’ southern distribution limit. North-Western Journal of Zoology 5: 429-433

Covaciu-Marcov SD, Cicort-Lucaciu AS, Dimancea N (2010) *Triturus dobrogicus* (Kiritzescu, 1903) in Caraş Severin county: status and conservation implications. Carpathian Journal of Earth and Environmental Sciences 5: 127-130

Covaciu-Marcov SD, Cicort-Lucaciu AS, Ferenţi S (2007) Some low altitude *Triturus montandonii* (Amphibia: Salamandridae) population records from Oaş region, North-Western Romania. North-Western Journal of Zoology 3: 109-114

Covaciu-Marcov SD, Cicort-Lucaciu AS, Gaceu O, Sas I, Ferenti S, Bogdan HV (2009) The herpetofauna of the south-western part of Mehedinţi County, Romania. North-Western Journal of Zoology 5: 142-164

Covaciu-Marcov SD, Cicort-Lucaciu AS, Ile RD, Paşcondea A, Vatamaniuc R (2007) Contributions to the study of the geographical distribution of the herpetofauna in the North-East area of Arad County, Romania. Herpetologica Romanica 1: 62-69

Covaciu-Marcov SD, Cicort-Lucaciu AS, Lazăr V, Szeibel N, Balaj L (2007) The herpetofauna of the lower hydrographical basin of Crişul Alb, the district of Arad (Romania). Oltenia, Studii şi Comunicări, Ştiinţele Naturii 23: 143-147

Covaciu-Marcov SD, Cicort-Lucaciu AS, Sas I, Bredet AM, Bogdan HV (2005) Herpetofauna from the basin of Mureş river in Arad county, Romania. Environment & Progress 5: 147-152

Covaciu-Marcov SD, Cicort-Lucaciu AS, Sas I, Groza MI, Bordaş I (2007) Contributions to the knowledge regarding the herpetofauna from the Maramureş county areas of ”Măgura Codrului”, Romania. Biharean Biologist 1: 50-56

Covaciu-Marcov SD, Cicort-Lucaciu AS, Sas I, Moşu AG, Toth B (2008) Contributions to the knowledge of the composition and geographical distribution of the Western Maramureş County Herpetofauna. Herpetologica Romanica 2: 27-36

Covaciu-Marcov SD, Cicort-Lucaciu AS, Sas I, Strugariu A, Cacuci P, Gherghel I (2008) Contributions to the knowledge regarding the composition and geographical distribution of the herpetofauna from Northern Moldavia (Suceava and Botosani Counties, Romania). North-Western Journal of Zoology 4: 25-47

Covaciu-Marcov SD, Ferenţi S (2008) About the presence of *Rana temporaria* species (Amphibia) at 150 m altitude in the Livada Forest (North-Western Romania). Oltenia, Studii şi Comunicări, Ştiinţele Naturii 24: 147-148

Covaciu-Marcov SD, Ghira I, Cicort-Lucaciu AS, Sas I, Strugariu A, Bogdan HV (2006) Contributions to knowledge regarding the geographical distribution of the herpetofauna of Dobrudja, Romania. North-Western Journal of Zoology 2: 88-125

Covaciu-Marcov SD, Ghira I, Sas I (2004) Contribuţii la studiul herpetofaunei zonei Oaşului (judeţul SM, România). Environment & Progress 2: 107-112

Covaciu-Marcov SD, Kovács I, Cicort-Lucaciu A, Sas I, Secare P (2009) Data upon the composition and the geografic distribution of the herpetofauna of the Almas-Agrij Depression (Salaj county, Romania). Oltenia, Studii şi Comunicări, Ştiinţele Naturii 25: 173-179

Covaciu-Marcov SD, Roşioru CL, Sas I (2011) Hot winters: new thermal habitats with frogs active in winter in north-western Romania. North-Western Journal of Zoology 7: 81-86

Covaciu-Marcov SD, Sas I, Cicort-Lucaciu A, Bogdan HV, Ardelean R (2006) The herpethofauna of the north-western region of Sălaj county, Romania. Studii şi Cercetări Ştiinţifice Universitatea din Bacău Seria Biologie 11: 85-90

Covaciu-Marcov SD, Sas I, Cicort-Lucaciu A, Bogdan HV, Groza M (2006) Contribuţii la cunoaşterea compoziţiei şi răspândirii herpetofaunei Moldovei dintre Siret şi Prut. Oltenia, Studii şi Comunicări, Ştiinţele Naturii 22: 242-247

Covaciu-Marcov SD, Sas I, Cicort-Lucaciu A, Kovács EH (2003) Notes upon the herpetofauna of the Northern area of the Botoşani county (Romania). Studii şi Cercetări Ştiinţifice Universitatea din Bacău Seria Biologie 8: 201-205

Covaciu-Marcov SD, Sas I, Cicort-Lucaciu A, Peter I, Bogdan HV (2005) Notes upon the herpetofauna of the south-west area of the county of Caraş-Severin, Romania. Revue Roumaine de Biologie Serie de Biologie Animale 50: 47-56

Covaciu-Marcov SD, Sas I, Cicort-Lucaciu AS (2007) Distribution of the pool frog *Pelophylax* (*Rana*) *lessonae* in North-Western Romania. Biota 8: 1-2

Covaciu–Marcov SD, Sas I, Cicort-Lucaciu AS, Kovács EH (2003) Notes upon the herpetofauna of the Northern area of Botosani County (Romania). Studii şi Cercetări Ştiinţifice Universitatea din Bacău Seria Biologie 8: 201-205

Covaciu-Marcov SD, Sas I, Cicort-Lucaciu AS, Kovács EH, Pintea C (2009) Herpetofauna of the Natural Reserves from Carei Plain: zoogeographical significance, ecology, statute and conservation. Carpathian Journal of Earth and Environmental Sciences 4: 69-80

Covaciu-Marcov SD, Sas I, Cupşa D (2008) On the presence of *Rana* (*Pelophylax*) *lessonae* in south-western Romania: distribution, biogeographical signification and status. North-Western Journal of Zoology 4: 129-133

Covaciu–Marcov SD, Sas I, Cupşa D, Kovacs EH, Groza M (2003) Contributions to the knowledge of the distribution of *Rana arvalis*–Nills. 1842 in the North–West region of Romania. Analele Universităţii din Oradea, Fascicula Biologie 10: 39-48

Covaciu-Marcov SD, Sas I, Cupşa D, Meleg G, Bud B (2003) Studii herpetologice în regiunea Munţilor Pădurea Craiului şi Plopişului (jud. Bihor, România). Analele Universităţii din Oradea, Fascicula Biologie 10: 81-95

Covaciu-Marcov SD, Sas I, Ilieş A (2010) *Pelophylax lessonae* (Amphibia) in Râul Doamnei, Argeş County, Romania. How have we arrived here? Biharean Biologist 4: 83-87

Covaciu-Marcov SD, Sas I, Kiss A, Bogdan HV, Cicort-Lucaciu AS (2006) The herpetofauna from the Teuz River hydrographic basin (Arad county, Romania). North-Western Journal of Zoology 2: 27-38

Covaciu-Marcov SD, Sas I, Lazar V, Szeibel N, Condure N (2008) The herpetofauna in the plain area from the western Satu Mare county, Romania. Oltenia, Studii şi Comunicări, Ştiinţele Naturii 24: 161-166

Covaciu-Marcov SD, Telcean I, Cupşa D, Cadleţ D, Zsurka R (2002) Contribuţii la studiul herpetofaunei din regiunea Marghita (jud. Bihor, România). Analele Universităţii din Oradea, Fascicula Biologie 9: 47-58

Covaciu-Marcov SD, Telcean I, Cupşa D, Sas I, Cicort-Lucaciu AS (2003) Contribuţii la cunoaşterea herpetofaunei regiunii bazinului hidrografic mediu şi inferior al Crişului Negru (jud. Bihor, România). Oltenia, Studii şi Comunicări, Ştiinţele Naturii 19: 189-194

Covaciu-Marcov SD, Telcean I, Sala G, Sas I, Cicort-Lucaciu A (2003) Contribuţii la cunoaşterea herpetofaunei regiunii Beiuş, jud. Bihor, România. Nymphaea Folia naturae Bihariae 30: 127-141

Cristea E, Cristea A, Demetriuc B (1972) Consideraţii cu privire la hrana naturală a broaştelor verzi de lac (*Rana ridibunda* Pall. şi *Rana esculenta* L.) din lunca şi Delta Dunării. Buletinul Institutului de Cercetari Piscicole 31: 19-23

Cruce M (1971) Contribuţii la studiul faunei herpetologice din Oltenia. Analele Universităţii din Craiova, III, Ştiinţe agricole şi biologice 3: 389-393

Csata E, Csata Z (1996) Răspândirea amfibienilor din partea centrală şi estică a Depresiunii Braşov. Acta Hargitensia 3: 49-60

Dehelean I, Ardelean G (2000) Herpetofauna zonei Firiza (Baia Mare). Satu-Mare, Studii şi Comunicări, Seria Ştiinţele Naturii I: 155-159

Dehelean I, Ardelean G (2000) Specii de amfibieni din Depresiunea Baia–Mare. Analele Universităţii din Oradea, Fascicula Biologie 7: 45-72

Demeter L, Hartel T, Cogălniceanu D (2006) Distribution and conservation status of amphibians in the Ciuc basin, Eastern Carpathians, Romania. Zeitschrift für Feldherpetologie Supplement 10: 217–224

Falcă M, Vasiliu-Oromulu L, Sanda V, Paucă-Comănescu M, Honciuc V, Maican S, Purice D, Dobre A, Stănescu M, Onete M, Biţă-Nicolae C, Matei B, Codrici I (2004) Ecosystemic characterization of some flooting ash forests from the Neajlov Holm (Giurgiu district). Proceedings of the Institute of Biology 6: 59-71

Fesci S (1969) Studiul preliminar al ecosistemelor din lacurile Doftana şi Brebu. Hidrobiologia 10: 221-230

Fesci S, Buza M (1973) Studiul geoecologic al rezervaţiilor din circurile glaciare ale muntilor Cindrel şi Şureanu. Ocrotirea naturii şi a mediului înconjurător 17: 203-208

Fuhn IE (1960) Amphibia. Fauna RPR, 14(1). Editura Academiei RPR, Bucureşti, 228

Fuhn IE (1963) Tritonul carpatic (*Triturus montandoni* Boulenger) în Munţii Făgăraşului. Natura 1: 78-79

Fuhn IE (1970) Amfibii şi reptile din zona viitorului lac de baraj de la Porţile de Fier. Studii şi cercetări de biologie Seria Zoologie 22: 321-332

Fuhn IE (1970) Aspecte ale situaţiei actuale a faunei din rezervaţia Pădurea Hagieni. Ocrotirea naturii şi a mediului înconjurător 14: 65-68

Fuhn IE (1971) Amfibii şi reptile din Delta Dunării. Peuce 1: 373-378

Fuhn IE (1974) Cercetari sinecologice cantitative asupra epigaionului Fetei Retezatului. Sargetia, Acta Musei Devensis, Series Scientia Naturae 10: 137-153

Fuhn IE (1975) Amphibia şi Reptilia. In: Ionescu M (Ed) Grupul de Cercetări Complexe "Porţile de Fier"-Seria Monografii ale Academiei RSR. Editura Academiei R.S.R., Bucuresti, 301-303

Fuhn IE, Cristurean I (1977) Situaţia actuală a rezervaţiei naturale Pădurea Hagieni. Ocrotirea naturii şi a mediului înconjurător 21: 103-110

Geormăneanu C (1975) The karyotype of the urodele amphibian *Triturus montandoni*. Revue Roumaine de Biologie Serie de Biologie Animale 20: 275-279

Gherghel I, Ile RD (2006) Contributions to the distribution of Amphibia, Caudata in Neamţ County, Romania. North-Western Journal of Zoology 2: 44-46

Gherghel I, Strugariu A (2007) Antropogenic impact upon the herpetofauna and the lake system from the future natural reserve from “Faurei Swamp”(Neamţ County, Romania). Analele Ştiinţifice ale Universităţii „Al I Cuza” Iaşi, seria Biologie animală 53: 175-179

Gherghel I, Strugariu A, Ambrosă IM, Zamfirescu SR (2012) Updated distribution of hybrids between *Lissotriton vulgaris* and *Lissotriton montandoni* (Amphibia: Caudata: Salamandridae) in Romania. Acta Herpetologica 7: 49-55

Gherghel I, Strugariu A, Ghira I (2010) On the presence of paedomorphosis in *Lissotriton vulgaris* (Amphibia: Salamandridae) from Danube Delta. Herpetologica Romanica 4: 62-64

Gherghel I, Strugariu A, Ghiurcă D, Roşu S, Huţuleac-Volosciuc MV (2007) The composition and distribution of the herpetofauna from the Valea Neagra river basin (Neamţ County, Romania. Herpetologica 1: 70-76

Gherghel I, Strugariu A, Glăvan T (2007) *Eremias arguta deserti* (Reptilia: Lacertidae) is not extinct from Romanian Moldavia. North-Western Journal of Zoology 3: 115-120

Gherghel I, Strugariu A, Pricop E, Zamfirescu SR (2008) The Northern Goşmani Mountains (Romania): An Important Herpetofaunal Area requiring urgent protection. Herpetologica Romanica 2: 51-54

Ghira I (1997) Herpetofauna of Crişul Repede/Sebes Körös and Barcău/Berettyó river basins. In: Sárkány-Kiss A, Hamar J (Eds) TISCIA Monograph series: The Criş/Körös rivers' Valleys. Department of Ecology, University of Szeged, Szeged-Arad, 353-361 pp.

Ghira I, Ghile P (1997) The herpetofauna of the River Someş/Szamos basin. In: Sárkány-Kiss A, Hamar J (Eds) TISCIA monograph series: The Someş/Szamos River Valley. Department of Ecology, University of Szeged, Arad-Szeged, 311-317 pp.

Ghira I, Marinescu IE, Domşa C (2003) Habitat preferences of different hybrid categories between *Bombina bombina* (L.) and *B. variegata* (L.) in Transylvanian plain. Studii şi Cercetări Ştiinţifice Universitatea din Bacău Seria Biologie 8: 211-215

Ghira I, Muresan D, Don MV (2004) Protecţia juridică a speciilor de amfibieni *Rana dalmatina* şi *Rana temporaria*. In: Statul de drept şi economia de piaţă în perspectiva integrării europene Sesiune internaţională de comunicări ştiinţifice. Editura Roprint, Cluj-Napoca, 539-545 pp.

Ghira I, Venczel M, Covaciu-Marcov SD, Mara G, Ghile P, Hartel T, Török Z, Farkas L, Rácz T, Farkas Z, Brad T (2002) Mapping of Transylvanian herpetofauna. Nymphaea Folia naturae Bihariae 29: 145-201

Ghiurcă D (2004) Estimarea cantitativă a populaţiilor de amfibieni din Parcul Natural Vânători Neamţ. Studii şi Cercetări Ştiinţifice Universitatea din Bacău Seria Biologie 19: 180-183

Ghiurcă D, Gherghel I (2008) Aspects concerning the herpetofauna in the city of Bacău (România): urban and periurban environments. Herpetologica Romanica 2: 13-19

Ghiurcă D, Gherghel I, Roşu G (2009) Contribution to knowledge of the distribution of herpetofauna in Tarcău Mountains (Romania). AES Bioflux 1: 73-79

Ghiurcă D, Munteanu A, Feneru F (2003) Some herpetological observations in Piatra Craiului National Park. Research in Piatra Craiului National Park 1: 273-274

Ghiurcă D, Rang G, Roşu S (2006) Preliminary data concerning the herpetofauna in Bacău county. Studii şi Cercetări Ştiinţifice Universitatea din Bacău Seria Biologie 11: 91-98

Ghiurcă D, Roşu S (2004) Contribuţii la studiul populaţiilor de amfibieni din zona Valea Budului – Luncani, judeţul Bacău. Studii şi Comunicări Muzeul Ştiinţele Naturii Bacău 19: 178-179

Ghiurcă D, Roşu S, Gherghel I (2005) Preliminary data concerning the herpetofauna in Neamţ County (Romania). Analele Universităţii din Oradea, Fascicula Biologie 12: 53-62

Groza MI, Lazăr V, Berinde DA, Pali IN (2007) Colour and morphological pattern data of two *Bombina bombina* populations from Dobrogea, Romania. Biharean Biologist 1: 5-9

Hartel T (2003) The breeding biology of the frog *Rana dalmatina* in Târnava Mare Valley, Romania. Russian Journal of Herpetology 10: 169-175

Iana S (1970) Noutăţi faunistice în ecosistemele Dobrogei de sud. Studii şi Comunicări Ocrotirea Naturii Suceava 1: 251-256

Ianc R, Cicort-Lucaciu AS, Ilieş D, Kovács EH (2012) Note on the presence of *Salamandra salamandra* (Amphibia) in caves from Padurea Craiului Mountains, Romania. North-Western Journal of Zoology 8: 202-204

Ifrim M (1972) Cercetări asupra speciei *Bufo viridis* L. (Anura, Amphibia) din bazinul mijlociu şi inferior al Bistriţei. Studii şi Cercetări Ştiinţifice Institutul Pedagogic Bacău Seria Ştiinţe Biologice 1: 39-49

Iftime A (2001) Observations on the amphibians and reptiles of the National Parks Semenic – Cheile Caraşului and Cheile Nerei – Beuşniţa (Romania). Travaux du Muséum National d'Histoire Naturelle "Grigore Antipa" 43: 323-332

Iftime A (2003) Contribution to the knowledge of the ichtiofauna and herpetofauna of Piatra Craiului National Park and its surrounding areas. Research in Piatra Craiului National Park 1: 267-272

Iftime A (2005) Herpetological observations in the Danube Floodplain sector in the Giurgiu county (Romania). Travaux du Muséum National d'Histoire Naturelle "Grigore Antipa" 48: 339-348

Iftime A (2005) New observations on the herpetofauna from Domogled-Valea Cernei National Park and Porţile de Fier Natural Park (Romania). Travaux du Muséum National d'Histoire Naturelle "Grigore Antipa" 48: 327-337

Iftime A (2005) Notes on the amphibians and reptiles in the region of Vidraru dam lake (southern cline of the Făgăraş Massif, Romania). Travaux du Muséum National d'Histoire Naturelle "Grigore Antipa" 48: 317-326

Iftime A, Gherghel I, Ghiurcă D (2008) Contribution to the knowledge on the herpetofauna of Bacău county (Romania). Travaux du Muséum National d'Histoire Naturelle "Grigore Antipa" 51: 243-253

Iftime A, Iftime O (2006) Herpetofauna masivelor forestiere continentale din sud-vestul Dobrogei. Situaţia actuală şi importanţa acesteia în conservarea habitatelor naturale. Delta Dunării 3: 141-152

Iftime A, Iftime O (2007) Some records of the herpetofauna of the Danube floodplain in the Balta Ialomiţei area (Romania). Travaux du Muséum National d'Histoire Naturelle "Grigore Antipa" 50: 273-281

Iftime A, Iftime O (2008) Observations on the herpetofauna of the Giurgiu county (Romania). Travaux du Muséum National d'Histoire Naturelle "Grigore Antipa" 51: 209–218

Iftime A, Iftime O (2010) Contributions to the knowledge of the herpetofauna of the Eastern Jiu and Upper Lotru drainage basins (Southern Carpathians, Romania). Travaux du Muséum National d'Histoire Naturelle "Grigore Antipa" 53: 273-286

Iftime A, Iftime O (2011) Note on the Herpetofauna of the Vâlcan Mountains and their Foothills (Southern Carpathians, Romania). Travaux du Muséum National d'Histoire Naturelle "Grigore Antipa" 54: 513-521

Iftime A, Iftime O (2012) New records of the Carpathian endemite, *Lissotriton montandoni* (Amphibia: Caudata: Salamandridae) at its southern distribution limit. Travaux du Muséum National d’Histoire Naturelle "Grigore Antipa" 55: 175–179

Iftime A, Petrescu AM, Iftime O (2008) Observations on the herpetofauna of the Mehedinţi karstic plateau (Mehedinţi and Gorj counties, Romania). Travaux du Muséum National d'Histoire Naturelle "Grigore Antipa" 51: 219-230

Ion I, Valenciuc N (1986) Studiul variabilităţii unor populaţii de salamandre. Analele Ştiinţifice ale Universităţii „Al I Cuza” Iaşi, seria Biologie animală 32: 114-117

Ionescu V, Miron I, Munteanu D, Simionescu V (1968) Vertebrate din bazinul montan al Bistriţei. Lucrările staţiunii de cercetări biologice, geologice şi geografice „Stejarul”, Pângaraţi 1: 375-437

Ionete L (1975) Notă herpetologică privind zona Fumureni-Vîlcea. Drobeta Studii şi Cercetări 1: 329-333

Jessat M (1998) Herpetologische Notizen aus Rumänien (1992 – 1997). Mauritiana 16: 598-600

Kiss JB (1985) Kétéltűek, hüllők (Amphibians, Reptiles). Editura Dacia, Cluj-Napoca, pp.

Kotenko T, Oţel V, Fedorchenko AA (1993) Herpetological investigations in the Danube Delta Biosphere Reserve in 1992. Analele Ştiinţifice ale Institutului Delta Dunării 2: 99-91

Krecsák L, Sike T, Sós T (2004) Distribution of the herpetofauna in the Lotrioara river basin, Sibiu district (Romania). Travaux du Muséum National d'Histoire Naturelle "Grigore Antipa" 47: 285-295

Lazăr V (2002) Condiţii optime de mediu pentru herpetofauna din sudul judeţului Dolj. IN: Muzeul Olteniei Craiova. Oltenia, Studii şi Comunicări, Ştiinţele Naturii 18: 199-201

Lazăr V (2004) Contribuţii la studiul tritonilor din herpetofauna judeţului Dolj. Oltenia, Studii şi Comunicări, Ştiinţele Naturii 20: 265-268

Lazăr V, Covaciu-Marcov SD, Sas I, Pusta C, Kovács EH (2005) The herpetofauna in the district of Dolj (Romania). Analele Ştiinţifice ale Universităţii „Al I Cuza” Iaşi, seria Biologie animală 51: 169-178

Mara G, Ghira I, Farkas L (1999) Preliminary report on herpetofauna of the upper and middle Olt river basin. Transylvanian Revue of Systematic Ecological Research 1: 169-181

Marcus A (1955) Observaţii asupra larvei de *Pelobates syriacus balcanicus* Karaman. Buletin Ştiinţific al Academiei RPR Secţiunea Ştiinţe Biologice Agricole Geologice Geografice 7: 785-789

Matei B (2002) Observations regarding the hibernation of amphibians in Caraorman marine levee (the Danube Delta). Proceedings of the Institute of Biology 4: 97-99

Micluţă H (1969) Contribuţii la cunoaşterea broaştei de mlaştină (*Rana arvalis*-Nilsson) (Notă preliminară). Buletinul Ştiinţific al Institutului Pedagogic Baia Mare, Seria B (Biologie Fizica Chimie Matematică) 1: 107-111

Micluţă H (1970) Note faunistice herpetologice din Judeţul Maramureş. Buletinul Ştiinţific al Institutului Pedagogic Baia Mare, Seria B (Biologie Fizica Chimie Matematică) 2: 39-42

Munteanu D (1973) Fauna de vertebrate a Masivului Ceahlău. Ocrotirea naturii şi a mediului înconjurător 17: 165-175

Niculescu F, Fuhn IE (1963) Cercetări asupra hranei broaştei de lac (*Rana r. ridibunda* Pall). Studii şi Cercetari Ştiinţifice, Academia Republicii Populare Romîne, Filiala Iaşi, (Biologie şi ştiinte agricole) 14: 193-211

Oprea A (2007) A proposal of a new natural park in the Eastern part of Romania. In: 1^st^ International Conference Environment – Natural Sciences-Food Industry in European Context. North University of Baia Mare, Chemistry-Biology Department, Baia Mare, 327-332 pp.

Oţel V (1992) Investigaţii herpetologice în rezervaţia Biosferei Delta Dunării (RBDD) în anul 1991. Analele Ştiinţifice ale Institutului Delta Dunării 1: 159-162

Oţel V (1997) Investigaţii herpetologice în zona munţilor Măcin şi podişul Babadagului. Analele Ştiinţifice ale Institutului Delta Dunării 6: 71-77

Oţel V (2000) The Red List of plant and animal species from the Danube Delta Biosphere Reserve Romania. Editura Aves, Tulcea, 132 pp.

Poliş R (1977) Răspîndirea broaştei de mlaştină (*Rana arvalis* Nilss) în valea Erului (judeţele Satu Mare şi Bihor). Nymphaea Folia naturae Bihariae 5: 417-425

Popescu M (1973) Contribuţii la cunoaşterea hranei adulţilor de *Rana ridibunda ridibunda* Pall. şi *Rana esculenta* L. din judeţul Tulcea. Peuce 3: 397-407

Popescu M (1977) Contribuţii la cunoaşterea broaştei roşii de pădure (*Rana dalmatina* Bonap. 1839) din judeţul Tulcea. Peuce 5: 155-163

Săhlean CT, Strugariu A, Zamfirescu SR, Pavel AG, Puşcaşu CM, Gherghel I (2008) A herpetological hotspot in peril: Anthropogenic impact upon the amphibian and reptile populations from the Băile Herculane tourist resort, Romania. Herpetologica Romanica 2: 37-46

Sălăgeanu G, Bavaru A, Fabritius K (1978) Rezervaţii, monumente şi frumuseţi ale naturii din judeţul Constanţa. Comitetul de Cultură şi Educaţie Socialistă al Judeţului Constanţa. Complexul Muzeal de Ştiinţe ale Naturii Constanţa, 1-102

Sas I (2009) Studii faunistice, ecologice şi fiziologice asupra complexului *Rana esculenta* din Nord-Vestul României. PhD thesis, Cluj-Napoca: Universitatea Babeş-Bolyai.

Sas I (2010) The *Pelophylax esculentus* complex in North-Western Romania: distribution of the population systems. North-Western Journal of Zoology 6: 294-308

Sas I, Covaciu-Marcov SD, Cupşa D, Schircanici A, Aszalós L (2003) Studiul spectrului trofic al unei populaţii de *Bombina bombina* (Linnaeus 1761) din zona Resighea (judeţul Satu–Mare, România). Oltenia, Studii şi Comunicări, Ştiinţele Naturii 20: 183-188

Sas I, Covaciu-Marcov SD, Demeter L, Cicort-Lucaciu AS, Strugariu A (2008) Distribution and status of the moor frog (*Rana arvalis*) in Romania. Zeitschrift für Feldherpetologie 13: 337–354

Sas I, Kovács EH, Covaciu-Marcov SD, Szatmári PM (2010) Southern distribution limit of *Pelophylax lessonae* and the LRE population system in Romania. Biharean Biologist 4: 185-188

Sas I, Roşioru CL, Covaciu-Marcov SD (2012) Note on eight new thermal habitats with winter-active amphibians in Western Romania. North-Western Journal of Zoology 8: 382-385

Schlüter U (2003) Die herpetofauna des NSG Pădurea Hagieni in Rumänien. Elaphe 11: 60-65

Schlüter U (2005) Die herpetofauna des Comorova-Waldes in Rumänien. Elaphe 13: 57-62

Sin G (1979) Cercetări asupra ecologiei broaştei *Rana ridibunda* Pallas 1771 din zona inundabilă a Dunării. PhD thesis, Bucureşti: Universitatea Bucureşti.

Sîrbu D (1976) Contribuţii la cunoaşterea hranei la *Bombina variegata* din împrejurimile oraşului Cluj Napoca-Napoca. Studia Universitatis Babeş-Bolyai Biologie 21: 65-70

Sós T (2005) Note preliminare privind distribuţia spaţială a herpetofaunei de pe Culmea Pricopanului din Parcul Naţional Munţii Măcin. Migrans 7: 8-10

Sós T (2007) Notes on distribution and current status of herpetofauna in the northern area of Braşov County (Romania). North-Western Journal of Zoology 3: 34-52

Sós T, Dároczi S (2008) Date suplimentare ale distribuţiei herpetofaunei în Dobrogea. Migrans 10: 2-5

Sós T, Szatmári B (2005) Săpăturile arheologice-capcane pentru amfibieni la Roşia Montana. Migrans 7: 8-9

Şova C (1969) Cercetări asupra variabilităţii unor populaţii de *Bombina variegata* din zona mijlocie a Carpaţilor Orientali. Studii şi Comunicări Muzeul de Ştiinţele Naturii Bacău 2: 193-229

Şova C (1970) Contribuţii la cunoaşterea faunei de amphibii din bazinul mijlociu al Siretului. Studii şi Cercetări Ştiinţifice Institutul Pedagogic Bacău Seria Ştiinţe Biologice 3: 101-119

Şova C (1972) Contribuţii la studiul ecologiei amfibiilor (ordinul Caudata, genul *Triturus*) din bazinul râului Siret. PhD thesis: Universitatea din Bucureşti.

Şova C, Cruce M (1969) Contribuţii privind variabilitatea populaţiilor de *Bombina variegata* (Amphibia, Discoglossidae). Analele Universităţii din Craiova, III, Ştiinţe agricole şi biologice 1: 485-499

Şova C, Tărăbuţă C (1963) Contribuţii la cunoaşterea faunei herpetologie din regiunea Bacău. I Amfibia. Comunicări de Zoologie Societatea de Ştiinţe Biologice 2: 221-224

Speybroeck, J. (2007) Herpetological trip to Romania (16^th^-28^th^ of May 2007). http://www.hylawerkgroep.be/jeroen/print.php?id=39 [accessed 1.12.2012]

Strugariu A, Gherghel I (2008) A preliminary report on the composition and distribution of the herpetofauna in the Lower Prut River Basin (Romania). North-Western Journal of Zoology 4: 49-69

Strugariu A, Gherghel I (2008) Spatial distribution of the herpetofauna from the upper and middle Moldova river basin (Romania). Travaux du Muséum National d’Histoire Naturelle "Grigore Antipa" 51: 231–241

Strugariu A, Gherghel I, Huţuleac-Volosciuc MV, Puşcaşu CM (2007) Preliminary aspects concerning the herpetofauna from urban and peri-urban environments from North-Eastern Romania: a case study in the city of Suceava. Herpetologica Romanica 1: 53-61

Strugariu A, Gherghel I, Huţuleac-Volosciuc MV, Săhlean TC, Sas I, Săhlean TC, Puşcaşu CM (2006) Preliminary data concerning the distribution of amphibian fauna in Suceava County (Romania). Analele Universităţii din Oradea, Fascicula Biologie 13: 39-47

Strugariu A, Gherghel I, Nicoară A, Huţuleac-Volosciuc MV, Moraru V, Mizeruş A (2009) A rapid survey of the herpetological fauna from Vaslui County (Romania) with the first record of the slow-worm (*Anguis fragilis*) in the region. Herpetologica Romanica 3: 25-30

Strugariu A, Sós T, Gherghel I, Ghira I, Săhlean TC, Puşcaşu CM, Huţuleac-Volosciuc MV (2008) Distribution and current status of the herpetofauna from the northern Măcin Mountains area (Tulcea County, Romania). Analele Ştiinţifice ale Universităţii „Al I Cuza” Iaşi, seria Biologie animală 54: 191-206

Strugariu A, Zamfirescu SR, Nicoară A, Gherghel I, Sas I, Puşcaşu CM, Bugeac T (2008) Preliminary data regarding the distribution and status of the herpetofauna in Iaşi County (Romania). North-Western Journal of Zoology 4: S1-S23

Stugren B (1966) Geographic variation and distribution of the Moor Frog, *Rana arvalis* Nilss. Annales Zoologici Fennici 3: 29-39

Stugren B (1966) Note faunistice herpetologice din Republica Socialistă România. Studii şi cercetări de biologie Seria Zoologie 18: 103-108

Stugren B, Iluţa L, Agadakos N, Markou K (1986) Variations des caractères biométriques de la grenouille rieuse, *Rana ridibunda* Pallas, dans le sud-est de l'Europe. Studia Universitatis Babeş-Bolyai Biologie 31: 30-34

Stugren B, Popovici N (1961) Note faunistice herpetologice din R.P.R. II. Studii şi cercetări de biologie Seria Zoologie 12: 229-234

Stugren B, Rusu R (1978) Ritmul circadian la buhaiul-de-baltă cu burta galbenă, *Bombina variegata* (L.) din România. Nymphaea Folia naturae Bihariae 6: 535-544

Stugren B, Vancea S (1968) Geographic variation of the yellow bellied toad (*Bombina variegata*)(L.) from the Carpathian Mountains of Romania and the USSR. Journal of Herpetology 2: 97-105

Székely P, Plaiaşu R, Tudor M, Cogălniceanu D (2009) A preliminary record list of amphibians in Dobrudja (Romania). Analele Ştiinţifice ale Universităţii „Al I Cuza” Iaşi, seria Biologie animală 50: 145-153

Székely P, Plaiaşu R, Tudor M, Cogălniceanu D (2009) The distribution and conservation status of amphibians in Dobrudja (Romania). Turkish Journal of Zoology 33: 147-156

Teleagă R (1976) Broaştele brune din Banat. Studiu sistemativ şi ecologic PhD thesis: Universitatea din Bucureşti.

Tesio C (1972) Modelul electroforetic al proteinelor sarcoplasmatice la speciile genului *Triturus* din România. Comunicări şi Referate ale Muzeului de Ştiinţele Naturii Ploieşti 1: 377-381

Török Z (1996) The protection of the herpetofauna in the Danube Delta. Rezumatele lucrărilor Simpozionului jubiliar "Rezervaţia naturală Codrii-25 de ani Realizări, probleme, perspective" Lozova, Republica Moldova: 124-126

Török Z (1997) Data on the actual status of the amphibian and reptile populations of the Someş river catchment area (Romania). Studii şi Cercetări Ştiinţifice Universitatea din Bacău Seria Biologie 2: 227-232

Török Z (1997) Data on the amphibians and reptiles from the Lăpuş river catchment area (Romania). Travaux du Muséum National d'Histoire Naturelle "Grigore Antipa" 39: 197-207

Török Z (1997) Data on the ecology of amphibians and reptiles from the sandy areas of the Razim-Sinoe Lagoonary System (Romania). Travaux du Muséum National d'Histoire Naturelle "Grigore Antipa" 37: 297-303

Török Z (1997) Herpetofauna bazinului Baia Mare. Analele Universităţii "Ovidius" Constanţa, Seria Biologie-Ecologie 1: 153-158

Török Z (1998) A quantitative approach to the road mortality in the herpetofauna from Grindul Chituc (Romania). Analele Ştiinţifice ale Institutului Delta Dunării 6: 159-166

Török Z (1998) Nişe ecologice spaţiale ale herpetofaunei de pe grindurile fluvio-maritime din complexul lagunar Razim-Sinoe (România). Acta Oecologica-Studii şi comunicări de ecologie şi protecţia mediului 5: 59-63

Török Z (1999) Data on the distribution of Amphibians and Reptiles in Gutâi and Igniş Mountains. Studii şi Cercetări Ştiinţifice Universitatea din Bacău Seria Biologie 4: 113-118

Török Z (1999) Note privind distribuţia spaţială a herpetofaunei în zona Culmii Pricopanului (Jud. Tulcea, România). Acta Oecologica-Studii şi comunicări de ecologie şi protecţia mediului 6: 57-62

Török Z (1999) Studies on the amphibians of the Someş/Szamos River-Valley. In: Sárkány-Kiss A, Hamar J (Eds) TISCIA monograph series – The Someş/Szamos River Valley. Department of Ecology, University of Szeged, Szeged-Arad, 303-309 pp.

Török Z (1999) Zonele umede din nord-vestul Dobrogei. Probleme de Ecologie Teoretică şi Aplicată din Romania-Direcţii Actuale. Editura Aves, Tulcea, 16 pp.

Török Z (2000) Date privind corologia şi protecţia amfibienilor şi reptilelor din Munţii Igniş. Satu-Mare, Studii şi Comunicări, Seria Ştiinţele Naturii 1: 160-170

Török Z (2001) Herpetological investigations in the lower Danube area (Calafat-Călăraşi sector). Studii şi Cercetări Ştiinţifice Universitatea din Bacău Seria Biologie 6: 115-119

Török Z (2002) Grindul Chituc. Probleme de Ecologie Teoretică şi Aplicată din Romania-Direcţii Actuale. Editura Aves, Tulcea, 16 pp.

Török Z (2004) Currently used techniques in assessing the *Rana ridibunda* stocks from the Danube Delta Biosphere Reserve (Romania). 1. Biomass calculation formulas. Analele Ştiinţifice ale Institutului Delta Dunării 10: 158-160

Török Z (2004) Data on the actual status of lake Plopu-Beibugeac (Tulcea county, Romania). Analele Ştiinţifice ale Institutului Delta Dunării 10: 71-80

Török Z (2004) Herpetological investigations in the Danube Delta Biosphere Reserve (Romania) in 2003. Analele Ştiinţifice ale Institutului Delta Dunării 10: 81-83

Török Z (2005) Amfibienii din Muntii Macin. Probleme de Ecologie Teoretică şi Aplicată din Romania-Direcţii Actuale. Editura Aves, Tulcea, 12 pp.

Tudor M, Crăciun N, Burlacu L (2004) Preliminary report on herpetofauna of the becoming National Parc „Jiului Gorge”. Oltenia, Studii şi Comunicări, Ştiinţele Naturii 20: 269-272

Vancea S, Boişteanu T, Malcoci-Chioreanu E (1972) Observaţii ecologice asupra pontelor de *Rana temporaria* din masivul Bîrnova (Iaşi). Studii şi Cercetări Ştiinţifice Universitatea din Bacău Seria Biologie 5: 233-242

Vancea S, Fuhn IE, Stugren B (1989) Sur la composition taxonomique du peuplement de grenouilles vertes (complexe *Rana esculenta* L) de Roumanie. Studia Universitatis Babeş-Bolyai Biologie 34: 69-77

Vasiliu-Suceveanu N (1968) Notă asupra răspîndirii metacercarului *Codonocephalus urnigerus* la *Rana ridibunda* din complexul Razelm-Sinoe. Buletinul Institutului de Cercetari Piscicole 27: 97-102

Vaucher CA (1975) Notes sur les vertébrés et les milieux naturels du Delta du Danube et de la Dobroudja. Presinge, Genève, 55 pp.

Wallis GP, Arntzen JW (1989) Mitochondrial-DNA variation in the crested newt superspecies: limited cytoplasmic gene flow among species. Evolution 43: 88-104

Zamfirescu SR (1999) New data regarding the amphibians' mapping in the Jijia river drainage basin. Analele Ştiinţifice ale Universităţii „Al I Cuza” Iaşi, seria Biologie animală 44/45: 147-151

Zamfirescu SR (1999) The mapping of herpetological fauna from the left side of Bicaz accumulation lake. Analele Ştiinţifice ale Universităţii „Al I Cuza” Iaşi, seria Biologie animală 44/45: 143-146

Zamfirescu SR (2000) New data regarding the amphibians mapping from Volovăţ, Ghireni, Başeu rivers drainage basin. Analele Ştiinţifice ale Universităţii „Al I Cuza” Iaşi, seria Biologie animală 47: 107-110

Zamfirescu SR, Strugariu A, Gherghel I, Zamfirescu O (2010) Sfântu Gheorghe (Tulcea, Romania): an important herpetological area. Analele Ştiinţifice ale Universităţii „Al I Cuza” Iaşi, seria Biologie animală 56: 119-128

Zamfirescu SR, Surugiu V (2001) The effect of the water salinity over the amphibians presence in Mangalia and Limanu lakes. Analele Ştiinţifice ale Universităţii „Al I Cuza” Iaşi, seria Biologie animală 47: 105-108

Zamfirescu SR, Zamfirescu O, Strugariu A, Gherghel I (2009) Herpetofauna of the meadows from the Site of Community Interest “The forest and the meadows from Mârzeşti” (Iaşi, Romania) and notes on habitats. Analele Ştiinţifice ale Universităţii „Al I Cuza” Iaşi, seria Biologie animală 55: 155-163
